# Multi-omics analysis reveals distinct gene regulatory mechanisms between primary and organoid-derived human hepatocytes

**DOI:** 10.1242/dmm.050883

**Published:** 2025-01-29

**Authors:** Haoyu Wu, Annie S. P. Yang, Suzan Stelloo, Floris J. M. Roos, René H. M. te Morsche, Anne H. Verkerk, Maria V. Luna-Velez, Laura Wingens, Johannes H. W. de Wilt, Robert W. Sauerwein, Klaas W. Mulder, Simon J. van Heeringen, Monique M. A. Verstegen, Luc J. W. van der Laan, Hendrik Marks, Richárd Bártfai

**Affiliations:** ^1^ Department of Molecular Biology, Faculty of Science, Radboud Institute for Molecular Life Science, Radboud University, Nijmegen 6525GA, The Netherlands; ^2^ Center for Infectious Diseases, Department of Medical Microbiology, Radboud University Medical Center, Nijmegen 6500HB, The Netherlands; ^3^ Department of Molecular Biology, Faculty of Science, Radboud Institute for Molecular Life Science, Oncode Institute, Radboud University, Nijmegen 6525GA, The Netherlands; ^4^ Department of Surgery, Erasmus University Medical Center Transplant Institute, University Medical Center Rotterdam, Rotterdam 3000CA, The Netherlands; ^5^ Department of Gastroenterology and Hepatology, Radboud Institute for Molecular Life Sciences, Radboud University Medical Center, Nijmegen 6500HB, The Netherlands; ^6^ Department of Molecular Developmental Biology, Faculty of Science, Radboud Institute for Molecular Life Sciences, Radboud University, Nijmegen 6525GA, The Netherlands; ^7^ Department of Surgery, Radboud University Medical Center, Nijmegen 6500HB, The Netherlands

**Keywords:** Intrahepatic cholangiocyte organoids, Primary human hepatocytes, scRNA-seq, Gene regulatory network, ELF3

## Abstract

Hepatic organoid cultures are a powerful model to study liver development and diseases *in vitro*. However, hepatocyte-like cells differentiated from these organoids remain immature compared to primary human hepatocytes (PHHs), which are the benchmark in the field. Here, we applied integrative single-cell transcriptome and chromatin accessibility analysis to reveal gene regulatory mechanisms underlying these differences. We found that, in mature human hepatocytes, activator protein 1 (AP-1) factors co-occupy regulatory regions with hepatocyte-specific transcription factors, including HNF4A, suggesting their potential cooperation in governing hepatic gene expression. Comparative analysis identified distinct transcription factor sets that are specifically active in either PHHs or intrahepatic cholangiocyte organoid (ICO)-derived human hepatocytes. ELF3 was one of the factors uniquely expressed in ICO-derived hepatocytes, and its expression negatively correlated with hepatic marker gene expression. Functional analysis further revealed that *ELF3* depletion increased the expression of key hepatic markers in ICO-derived hepatocytes. Our integrative analysis provides insights into the transcriptional regulatory networks of PHHs and hepatic organoids, thereby informing future strategies for developing improved hepatic models.

## Teaser

**Editor's choice:** Through comparative epigenome and transcriptome analysis, we identified key gene regulatory differences between primary and intrahepatic cholangiocyte organoid-derived human hepatocytes, which will support the development of improved hepatic models.

## INTRODUCTION

The human adult liver is a complex and regenerative organ that is composed of parenchymal and non-parenchymal cell types. Primary human hepatocytes (PHHs), which make up more than 80% of the total liver mass, are essential for a range of activities performed by the liver, such as glycogen storage, lipid/carbohydrate metabolism, and inactivation of exogenous and endogenous compounds (Hansel et al., 2015). Fully matured PHHs are functionally heterogeneous along the porto-central axis of the liver lobule, known as liver zonation. PHHs stay in a quiescent state but can re-enter the cell cycle to start proliferation following damage (Fausto, 1997; Furchtgott et al., 2009). Hepatocyte regeneration is mainly separated into three phases: the priming phase, the proliferation phase and the termination phase (Gilgenkrantz and Collin de l'Hortet, 2018). Studies on liver regeneration have mainly been performed using mouse models, investigating the origin of hepatocyte regeneration. However, this process remains less well characterized in humans owing to the lack of a proper model.

PHH culture is currently the main *in vitro* model for studies regarding hepatocytes, such as for prediction of drug toxicity, modeling liver diseases and studies towards liver transplantation. However, the utilization of PHHs is limited owing to difficulty in maintaining their functional and cellular identity, and the lack of their capacity to extensively proliferate *in vitro*. Therefore, the past decades have seen numerous studies aiming to solve these challenges, and currently one of the most promising approaches is the use of 3D *in vitro* cultures, liver organoids (Prior et al., 2019). Different from cells in 2D culture systems, organoids form a 3D structure by self-organization, display high levels of genetic stability, and recapitulate structural and functional aspects of the organs *in vivo* (Huch et al., 2015; Marsee et al., 2021). Human liver organoids can be generated from various sources, including induced pluripotent stem cells, or from fetal and adult primary tissues (Prior et al., 2019). Depending on their origin, organoids display distinct characteristics, functions and differentiating/self-renewing capacities (Prior et al., 2019). For example, the recently generated human adult hepatocyte organoids display both progenitor and mature hepatic features (Huch et al., 2015; Marsee et al., 2021). However, the establishment and maintenance of these hepatocyte-derived organoids remain challenging (Hu et al., 2018; Peng et al., 2021). Human intrahepatic cholangiocyte organoids (ICOs), generated from EPCAM-positive bile duct cells and maintained in expansion medium (EM; i.e. with the WNT pathway activated), on the other hand, are highly proliferative and can be differentiated toward hepatocyte-like cells when cultured in hepatocyte differentiation medium (DM) (Huch et al., 2015; Marsee et al., 2021). This makes DM-ICOs an attractive model for long-term maintenance of liver cells *in vitro*. However, hepatocyte-like cells differentiated from ICOs remain immature compared to PHHs (Huch et al., 2015), which hampered the usage of this model in therapeutics and fundamental studies (Chen et al., 2018). Thus, there is an urgent need for further improvement of functional ICO-derived hepatocyte differentiation *in vitro*, which would highly benefit from further understanding of the gene regulatory mechanisms underlying hepatocyte differentiation and maturation.

Cell identity is governed by gene expression patterns, which are controlled by transcription factors (TFs), epigenetic modifiers and chromatin remodelers in gene regulatory networks. In the liver, studies have shown essential roles for hepatic TFs in controlling multiple biological processes, and dysregulation of hepatic TFs has been associated with developmental defects, failure of liver regeneration and various liver diseases (Lee et al., 2005; Pepe-Mooney et al., 2019; Cebola, 2020). Therefore, a better understanding of how TFs orchestrate gene expression in hepatocytes would provide important insights into the molecular mechanism of hepatocyte maturation both *in vivo* and *in vitro*. TFs required for cell-fate decision during liver development have been explored mostly in mouse models or during *in vitro* pluripotent stem cell differentiation. For example, hepatocyte nuclear factors (HNFs), such as HNF4A, FOXA1 and FOXA2 (also known as HNF3A and HNF3B, respectively), together with GATA-binding factor (GATA)4/6 and CCAAT enhancer binding protein (CEBP)A/B, are liver-enriched TFs that are important for normal liver development (Tachmatzidi et al., 2021). However, substantial variability between mouse and human limits the use of the mouse models for understanding human liver biology and pathogenesis. Moreover, the gap between human liver organoids and mature hepatocytes has yet to be explored. As such, it remains elusive, thus far, how TFs are interacting to orchestrate the gene regulatory network of human hepatocytes, and how the gene regulatory mechanisms in ICO-derived hepatocyte-like cells (DM-ICOs) differ from those in PHHs.

In this study, we combined RNA sequencing (RNA-seq) at single-cell resolution (scRNA-seq) and assay for transposase-accessible chromatin using sequencing (ATAC-seq) to dissect the gene regulatory mechanisms in PHHs and ICOs. Using an optimized two-step perfusion protocol, we isolated a nearly pure population of PHHs. scRNA-seq analysis displayed that, even after tissue dissociation, the heterogeneity between PHHs is driven by the liver zonation. Integrative multi-omics analysis in PHHs suggests that activator protein 1 (AP-1), a dimeric TF composed of JUN, FOS or activating transcription factor (ATF), which is essential for hepatic regeneration, also contributes to the establishment of the hepatic gene expression program in conjunction with typical liver-specific TFs, including HNF4A. Comparison of the gene regulatory modules revealed distinct TF sets required for PHHs and ICOs, whereas, surprisingly, gene regulatory TFs and chromatin accessibility between EM-ICOs and DM-ICOs only showed subtle differences. Further studies on ELF3, the most prominent TF that is specifically expressed in ICOs, showed that depletion of *ELF3* during hepatocyte differentiation of ICOs promotes the expression of mature hepatocyte markers, suggesting that ELF3 functions as a barrier of hepatocyte differentiation from ICOs.

## RESULTS

### Liver zonation is the main driver of heterogeneity among PHHs

**Fig. 1.**
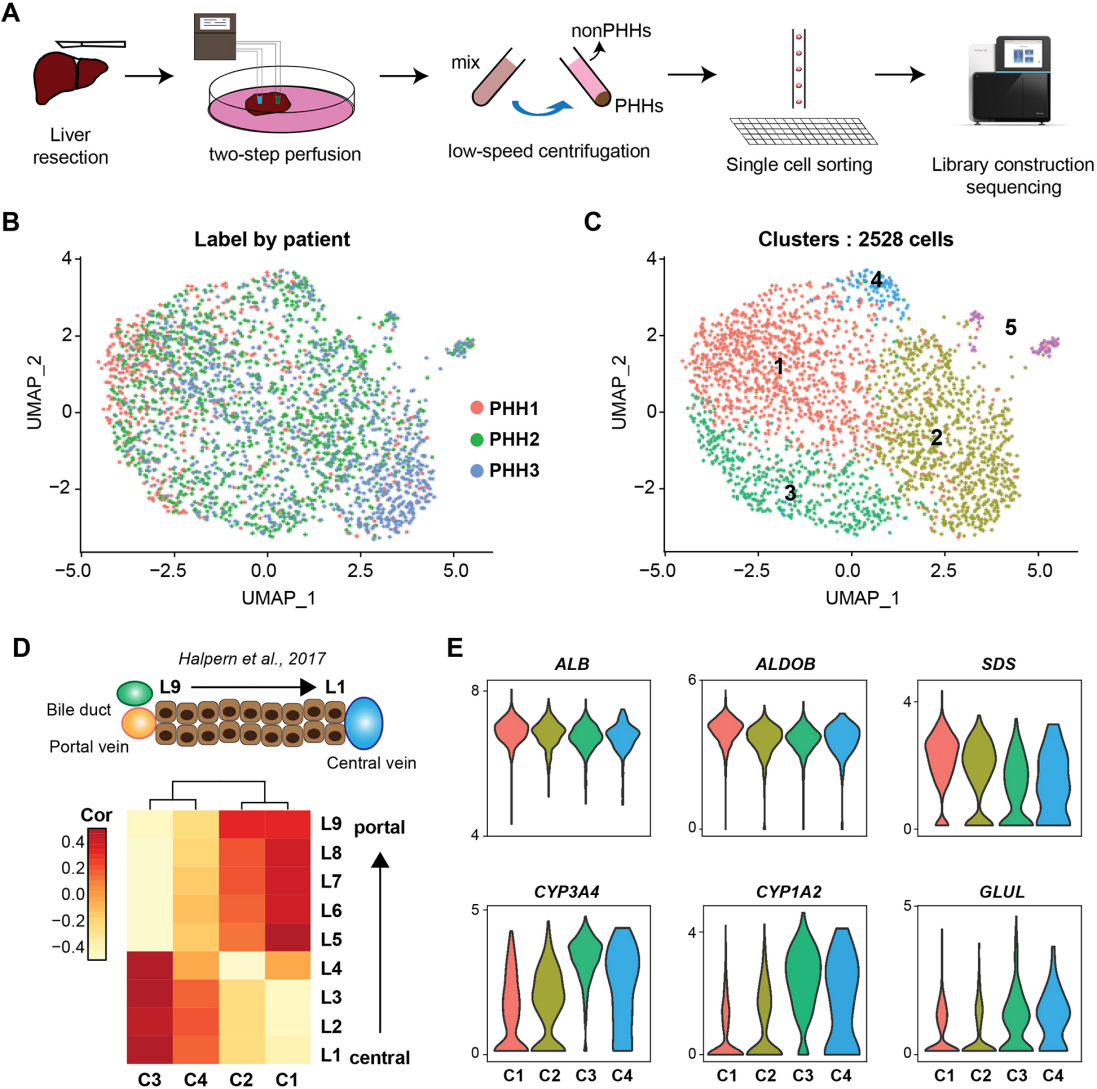
**Single-cell RNA-sequencing (scRNA-seq) analysis shows that liver zonation is the main driver of heterogeneity observed in primary human hepatocytes (PHHs).** (A) Overview of single-cell isolation from liver segments following a modified two-step perfusion protocol and fluorescence-activated cell sorting workflow. (B,C) Uniform manifold approximation and projection (UMAP) plots of scRNA-seq data colored by donors (left) and cluster number (right). (D) Heatmap showing the correlation of selected zonal genes between hepatocyte clusters identified in this study and the nine layers of mouse liver lobule (L1, central; L9 periportal). Generated using data from Halpern et al. (2017). (E) Violin plots showing expression of representative zonal markers (periportal: *ALB*, *ALDOB* and *SDS*; pericentral: *CYP3A4*, *CYP1A2* and *GLUL*) across hepatocyte clusters.

Previous scRNA-seq datasets were mainly generated from the whole liver, aiming to recapitulate the full complexity of cell types within the liver tissue (MacParland et al., 2018; Aizarani et al., 2019). However, unbiased isolation of all cell types leads to a limited and inconsistent proportion of PHHs from each donor/sample, hence compromising in-depth analysis of PHHs. Therefore, we applied a two-step perfusion isolation approach to obtain pure human hepatocytes for scRNA-seq analysis (Yang et al., 2021; see Materials and Methods, Fig. 1A). We obtained 2528 high-quality cells from three adult livers, and identified five distinct cell clusters after normalization and batch correction (Fig. 1B,C; Fig. S1A-C). Underscoring the efficiency of our PHH isolation protocol, most of the cells displayed high expression levels of matured hepatocyte markers, but low or no expression of liver progenitor cell- or cholangiocyte-associated genes (Fig. S1D). In fact, only ∼2% of the cells (cluster 5) represented other cell types and showed expression of well-known markers for endothelial/epithelial and immune cells (*DCN*, *CD74*, *ZEB2* and *TCF4*; Fig. S1D,E, Table S1), which we excluded from further analysis.

Genes most prominently enriched in cluster 1 included *SDS* and *ASS1*, while *CYP1A2*, *CYP3A4* and *GLUL* were enriched in cluster 3 (Fig. S1E), all representing markers known to be associated with liver zonation. Indeed, when comparing our data with zonally resolved expression data from mouse, we observed a clear zonal pattern, with clusters 1 and 2 displaying more similarities to the periportal zone and clusters 3 and 4 sharing pericentral features (Halpern et al., 2017; MacParland et al., 2018) (Fig. 1D,E; Fig. S2A). Altogether, our data further strengthen that porto-central zonation is the primary driver of heterogeneity amongst PHHs even after dissociation of the liver tissue.

### Gene regulatory network analysis identifies a potential crosstalk between AP-1 and liver-specific TFs

**Fig. 2.**
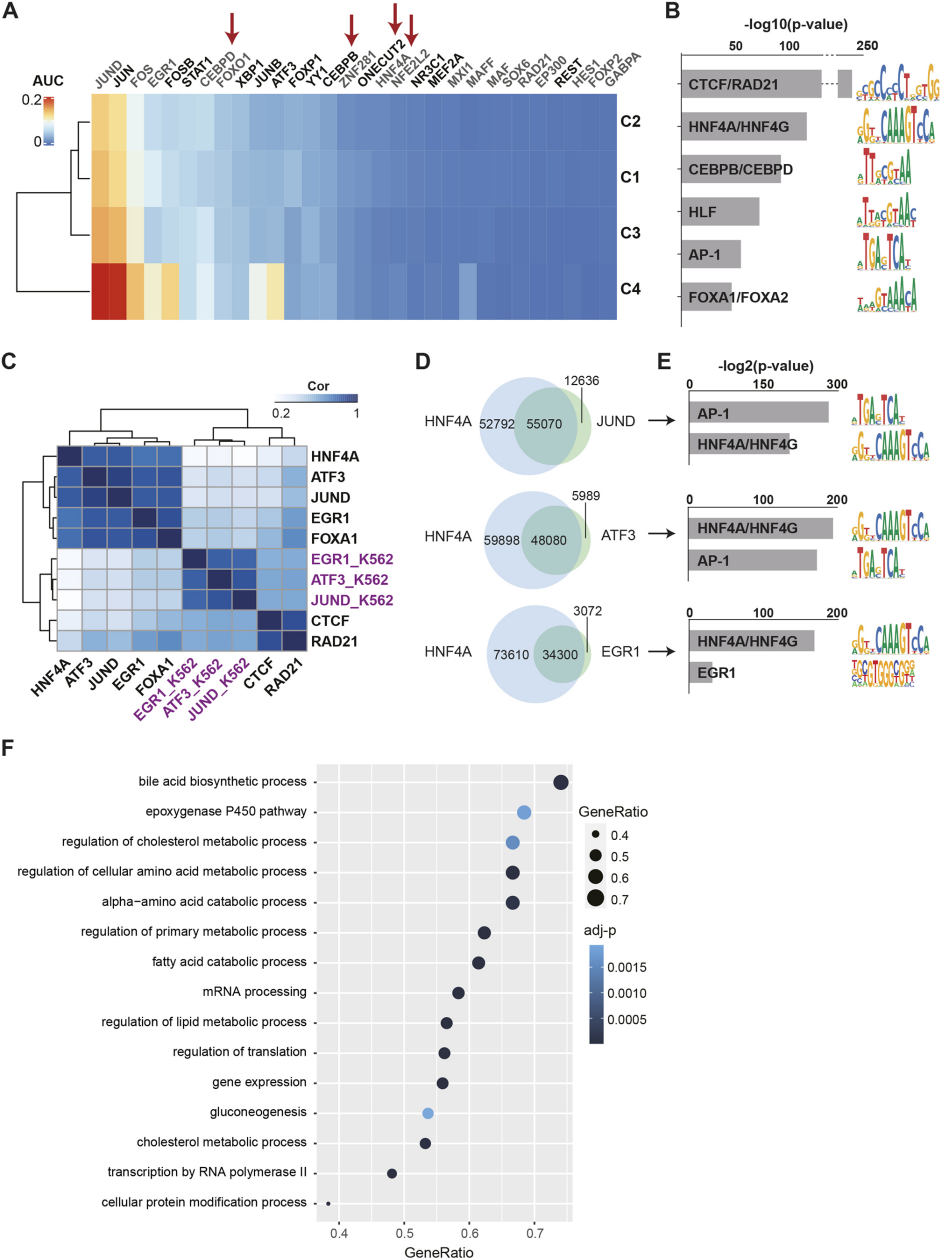
**Gene regulatory network analysis identifies co-occupancy of AP-1 and liver-specific transcription factors in PHHs.** (A) Heatmap of enrichment of regulons [shown as average area under the recovery curve (AUC) score] identified in the four hepatocyte clusters. Regulons in gray font represent regulons in which non-direct motifs were used for prediction of potential targets of the transcription factors (TFs) and co-regulators; regulons in black font represent regulons in which direct motifs were used for prediction of potential targets of the TFs and co-regulators (see Materials and Methods). Red arrows point at the well-known hepatic TFs. (B) List of the top 6 motifs enriched in assay for transposase-accessible chromatin using sequencing (ATAC-seq) peaks in PHHs. Motifs are ranked by −log10(*P*-value). (C) Heatmap of Spearman correlation matrix of normalized TF binding intensities in open chromatin regions in PHHs as identified by ATAC-seq. TFs in black font are from liver; TFs in purple font are from K562 lymphoblast cell line (as control). (D) Venn diagram showing the intersection between binding sites of HNF4A and those of JUND, ATF3 and EGR1, respectively. (E) Motifs enriched in overlapping chromatin immunoprecipitation sequencing (ChIP-seq) peak regions of HNF4A and those of JUND, ATF3 and EGR1 (as in D), respectively. Motifs were ranked based on −log10(*P*-value). (F) Dot plot of Gene Ontology (GO) analysis (biological process), showing the selected significant [adjusted *P* (adj-*P*)<0.05] pathways enriched for the HNF4A and JUND co-bound genes. Circle color indicates the significance of the enrichment, while the size represents the gene ratio.

Gene regulatory networks determine cell identity and function. To identify the key regulatory factors in the primary hepatocytes, we applied single-cell regulatory network inference and clustering (SCENIC). SCENIC identifies potential master TFs and co-regulators as well as their downstream target genes (called regulons) by integrating co-expression modules between TFs and their target genes and *cis*-regulatory motif enrichment at these target gene loci. Using the PHHs as present in cluster 1 to 4, we detected, in total, 63 regulons with relatively little difference between the four clusters (Fig. S3A). We identified HNF4A, CEBPB, CEBPD and ONECUT2 regulons, of which the corresponding TFs are well known and essential for liver development (Margagliotti et al., 2007; Tachmatzidi et al., 2021) (Fig. 2A). FOXO1 and FOXP1 TFs regulate hepatic glucose homeostasis through response to insulin (Tikhanovich et al., 2013; Zou et al., 2015). In general, we found that the regulons of AP-1 TFs such as JUND, JUN, JUNB, FOS, FOSB, EGR1 and ATF3, classically associated with cellular proliferation and oncogenesis, were highly active in isolated primary hepatocytes, which was even more pronounced in cluster 4 (Fig. 2A). Taking a closer look, we found that genes enriched in cluster 4 included not only AP-1 genes, but also *TNFAIP3*, *GADD45B*, *SERPINE1* and *G0S2* (Fig. S3B), known to be upregulated immediately in the priming phase of mouse hepatocytes as an initial step in hepatic regeneration (Su et al., 2002; Li et al., 2014). Thus, cells in cluster 4 might represent the elusive human hepatocytes in their priming phase.

In parallel to our scRNA-seq analysis, we carried out chromatin accessibility profiling (ATAC-seq) on the PHHs isolated from two different donors and performed motif analysis on the open chromatin regions. Here, we detected enrichment of CTCF/RAD21, HNF4, CEBP, AP-1 and FOXA1 motifs in the accessible chromatin regions (Fig. 2B), in line with the SCENIC scRNA-seq analysis. Overall, our analysis indicates that, besides in liver regeneration, AP-1 TFs might play a regulatory role in mature hepatocytes.

Notably, it has been reported that tissue dissociation may evoke cellular stress and gene expression changes, including altered expression of AP-1 factors. To investigate this, we compared our PHH scRNA-seq data with published single-nuclei RNA-seq (snRNA-seq) data (Brazovskaja et al., 2024), which do not require enzymatic dissociation and hence might closer reflect genuine gene expression levels in the liver (Fig. S2B). Although we indeed observed some quantitative differences in mRNA levels of hepatocyte marker genes and TFs between these two technologies, we did not observe strong activation of genes related to stress pathways in our dataset. Importantly, expression of AP-1 factors was detected in both approaches, suggesting that the expression of AP-1 factors is not the sole consequence of tissue dissociation. To further confirm the roles of AP-1 TFs in the tissue of origin, we re-analyzed published JUND, ATF3, EGR1 FOXA1, HNF4A, CTCF and the cohesion ring component RAD21 chromatin immunoprecipitation sequencing (ChIP-seq) datasets from human liver tissue [from the Encyclopedia of DNA Elements (ENCODE)], including JUND, ATF3 and EGR1 ChIP-seq datasets from the K562 lymphoblast cell line as controls (from ENCODE) and integrated these with our ATAC-seq data. In agreement with the motif analysis, high enrichment of HNF4A and AP-1 (EGR1, JUND and ATF3) TF occupancies were found in the liver tissue in overlap with the ATAC peaks in PHHs (Fig. S3C,D). We evaluated and compared genome-wide binding profiles of the selected TFs in accessible regions in the PHHs by quantification of the ChIP-seq signals from the liver tissue within the PHH ATAC-seq peak regions. Spearman correlation analysis of TF binding within open chromatin regions revealed that AP-1 TF binding profiles displayed strong correlation with HNF4A and FOXA1 binding profiles in the liver, but not in K562 lymphoblast cells (Fig. 2C; Fig. S3D). Next, we identified ChIP-seq peaks for HNF4A, JUND, ATF3 and EGR1 to determine the overlap in occupancies. This analysis showed that the three AP-1 factor binding sites are highly enriched in HNF4A peak regions (Fig. S3C). We observed that 81% (55,070 of 67,706), 89% (48,080 of 54,069) and 92% (34,300 of 37,372) of the JUND, ATF3 and EGR1 peaks, respectively, were intersecting with HNF4A binding sites (Fig. 2D). Accordingly, motif analysis revealed significant enrichment for the HNF4A motif at JUND, ATF3 and, to a lesser extent, EGR1 motifs in peak regions (Fig. 2E). To elucidate the potential regulatory roles of AP-1 TFs in hepatocytes, we next focused on genes that are targeted by HNF4A and JUND. Among 7716 genes with detectable levels of transcript abundance in our PHH scRNA-seq data, we observed 5137 genes that are bound by both HNF4A and JUND compared to target genes bound by either HNF4A (743 genes) or JUND (62 genes) only (Fig. S3E). The HNF4A/JUND co-occupied genes showed enrichment of Gene Ontology (GO) categories associated with fundamental biological pathways, including gene expression, RNA processing, protein modification and transcription regulation, as well as multiple metabolic processes related to lipid, fatty acid, bile acid, epoxygenase P450 pathway and gluconeogenesis (Fig. 2F). Altogether, our analysis suggests that AP-1 TFs, by co-occupying regulatory regions with liver-specific TFs, are likely to be associated with fundamental cellular and liver-specific functions in hepatocytes. Furthermore, AP-1 TFs seem to be prominent features of regenerating human hepatocytes in their priming phase, as shown in mice (Su et al., 2002).

### Comparative analysis uncovers distinct TFs required in PHHs and ICOs

Previous studies demonstrated that ICOs have the potential to differentiate towards the hepatocyte lineage by culturing the ICOs in DM (Huch et al., 2015; Verstegen et al., 2020). However, these organoid-derived hepatocyte-like cells are not fully matured compared to primary hepatocytes. To identify the gene regulatory differences among ICOs in different culture conditions as well as between ICOs and PHHs that could explain the above observations, we carried out scRNA-seq on the ICOs cultured in either EM or DM (EM-ICOs versus DM-ICOs). Differentiation of liver organoids was performed with three organoid lines derived from different donors, as previously described (Broutier et al., 2016) (Fig. S4A). In total, we generated single-cell transcriptomic profiles with high quality [unique molecular identifiers (UMIs)>1000 and percentage of mitochondrial gene expression (mt%)<50%] from 1268 ICO cells. Uniform manifold approximation and projection (UMAP) indicated that cells are clustered based on culture conditions (Fig. S4B). Our ICOs were of regular sizes of a few millimeters (Verstegen et al., 2020) and expressed only very low levels of necroptosis genes such as *RIPK3*, *RIPK1* and *TLR3*, indicating that their gene expression pattern is not majorly affected by necrosis, which could occur in the (hollow) middle of these organoids owing to oxygen and nutrient deprivation (Fig. S4A-C). Analysis of cell-cycle-related genes showed more DM-ICO cells than EM-ICO cells in G1 phase, suggesting that DM-ICOs are, as expected, less proliferative (Fig. S4D). In line with previous reports (Huch et al., 2015; Verstegen et al., 2020), we detected downregulation of the WNT target gene *LGR5* and progenitor/cholangiocyte marker *SOX9*, and upregulation of hepatocyte markers (*ALB*, *CYP3A4*, *GLUL* and *GC*) (Fig. S4E-G). Notably, the DM-ICOs did not show a clear gene expression signature associated with either the pericentral or periportal zone after differentiation, and hence organoids do not recapitulate this important aspect of hepatocyte biology (Fig. S4H). Expression of cholangiocyte markers (*TFF1*, *TFF3* and *MUC5B*) in DM-ICOs decreased compared to that in EM-ICOs, while other cholangiocyte markers such as *EPCAM*, *KRT7* and *KRT19* remained largely similar (Fig. S4G), indicating that ICOs under hepatocyte differentiation condition still retain cholangiocyte features. Next, to better understand the transcriptome of EM-ICOs and DM-ICOs, we built a Random Forest classifier based on the published human liver scRNA-seq data (MacParland et al., 2018; Andrews et al., 2022), and evaluated the similarities between ICOs and the *in vivo* human liver cells (see Materials and Methods). We found that ICOs, independent of culture conditions, substantially resembled human cholangiocytes (Fig. S4I). Therefore, our analyses indicate that ICOs under expansion and hepatocyte differentiation conditions largely retain cholangiocyte features.

**Fig. 3.**
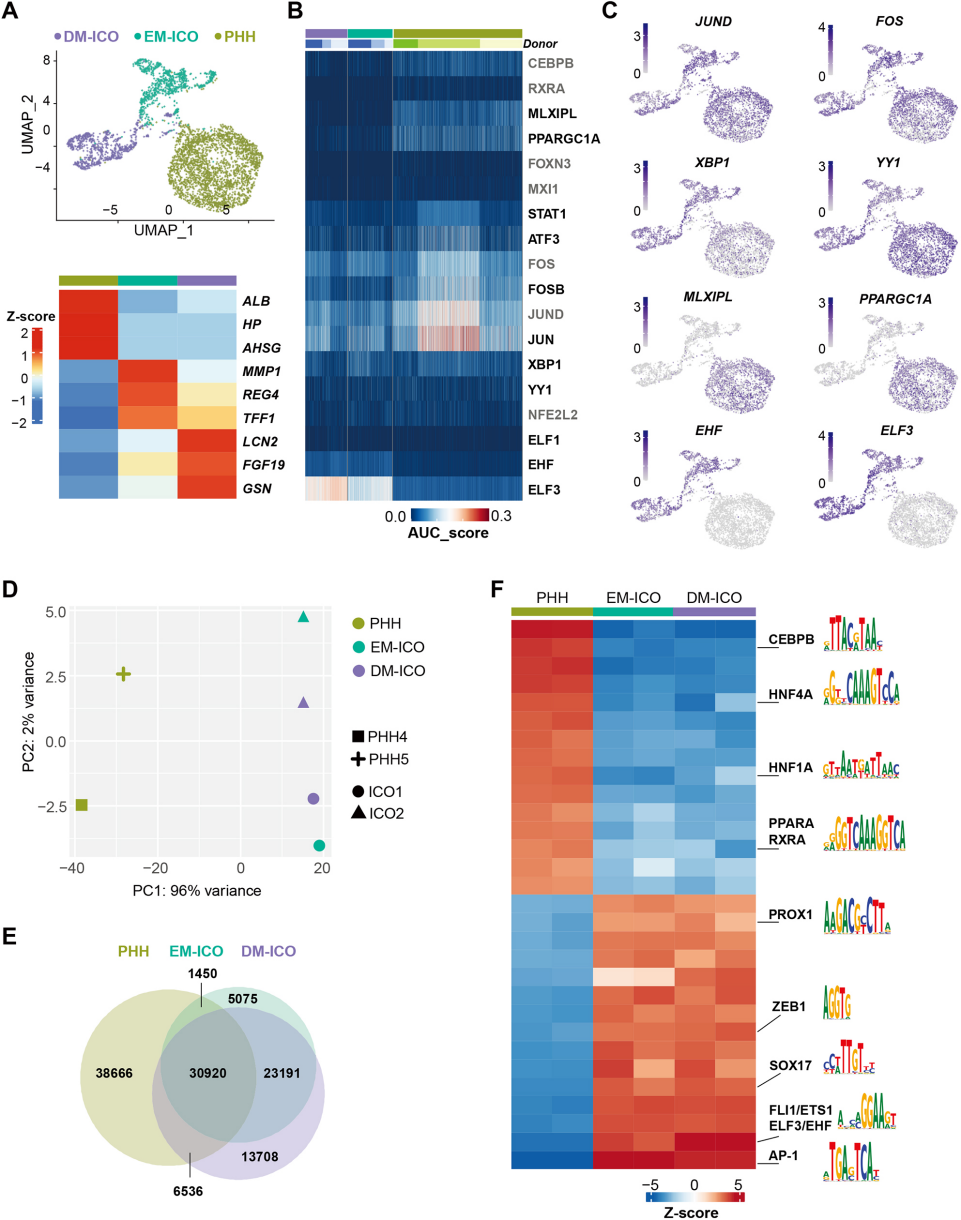
**Integrative analysis of scRNA-seq and ATAC-seq identifies distinct TFs required in PHHs and intrahepatic cholangiocyte organoids (ICOs).** (A) UMAP of scRNA-seq data from PHHs, ICOs maintained in expansion medium (EM-ICOs) and ICOs maintained in differentiation medium (DM-ICOs) (top), and heatmap showing the expression of the top 3 marker genes in *z*-score format for each cell type (bottom). Cells are colored based on the cell type. (B) Heatmap of the AUC score of selected regulons in each single cell from PHHs and ICOs as analyzed by single-cell regulatory network inference and clustering (SCENIC). Colors of each cell type match those of A. (C) Expression UMAPs of *JUND*, *FOS*, *XBP1*, *YY1*, *MLXIPL*, *PPARGC1A*, *EHF* and *ELF3*. Data were normalized to sequencing depth and are shown in log2 format. (D) Principal component (PC) analysis plot showing the similarities of the genome-wide chromatin state between PHHs, EM-ICOs and DM-ICOs. Biological replicates are shown in different shapes, and cell types are shown in different colors as in A. (E) Venn diagram showing the intersection between ATAC peaks of PHHs, EM-ICOs and DM-ICOs. (F) Heatmap showing motif enrichment analysis between PHHs, EM-ICOs and DM-ICOs in *z*-score format using Gimme Motif maelstrom. Motifs of interest are shown on the right.

To gain insights into the gene regulatory mechanisms that could explain the lack of full maturation of ICOs, we performed integrated analysis of the ICO and PHH datasets. We found that cells were clearly separated based on the different cell types with clear distinct transcriptional signatures of PHHs and ICOs (Fig. 3A). Next, we used SCENIC to identify master regulators in hepatocytes and ICOs from both conditions. In total, 56 regulons were found, some of which were enriched across different cell types, such as AP-1 TFs STAT1, YY1, XBP1 and NFE2L2. Some TFs were specific to either PHHs or ICOs (Fig. 3B). For example, MLX interacting protein like (MLXIPL; also known as ChREBP) is a glucose-sensitive TF that is crucial for glucose metabolisms and *de novo* lipogenesis (Iizuka et al., 2004). Peroxisome proliferator-activated receptor gamma coactivator 1-alpha (PPARGC1A) is required for nutrient metabolism in the liver (Lin et al., 2005; Besse-Patin et al., 2019). Both (co)factors are associated with metabolism and are more enriched in PHHs, in line with the fact that PHHs display a more physiologically relevant gene expression pattern than that of ICOs. On the other hand, we identified ELF3 and EHF as main regulons in ICOs (EM and DM) that are absent in PHHs. In line with the enrichment of regulons, expression levels of these TFs also displayed similar trends between PHHs and ICOs (Fig. 3C).

To explore the chromatin landscape in ICOs, we performed bulk ATAC-seq on two ICO lines cultured in EM and DM, and examined the divergence of chromatin accessibility profiles between PHHs, EM-ICOs and DM-ICOs. Overall, we observed little difference between the biological replicates and culture conditions of ICOs (EM and DM) but substantial difference between PHHs and organoids (Fig. 3D; Fig. S5A-C). When comparing ATAC-seq peaks across different samples, more shared ATAC-seq peaks were found among ICOs than those between PHHs and ICOs (Fig. 3E), in agreement with the correlation analysis. The ATAC-seq signal over the top 2000 unique peaks from each sample also showed clear differences between PHHs and ICOs (Fig. S5B). Therefore, our analysis highlights a distinct chromatin landscape between PHHs and ICOs.

TFs bind to specific DNA motifs to regulate gene expression. Thus, we performed motif analysis using GimmeMotifs to determine which TFs were potentially associated with open chromatin regions from EM-ICOs and DM-ICOs. As expected, ICOs in both EM and DM are enriched for similar motif sets, including, but not limited to, CTCF, BACH2, ELF3, JUN, HNF4A and FOXA1, some of which were also enriched in PHH open chromatin regions (Fig. 2B; Table S2). We next applied Gimme maelstrom to analyze differentially enriched motifs on the unique peaks from PHHs, EM-ICOs and DM-ICOs (Fig. 3F). In agreement with our previous observation from SCENIC analysis, we detected high enrichment of TF motifs, such as CEBPB, RXRA, PPARG, HNF4A and HNF1A in PHHs. Furthermore, motifs of ELF3, EHF, SOX17 and PROX1 were, again, more enriched in ICOs. Taken together, our data showed that distinct sets of TFs are required for PHHs and *in vitro* ICOs and identified ELF3 as a TF strongly associated with ICOs.

### *ELF3* depletion promotes hepatic differentiation of ICOs *in vitro*

**Fig. 4.**
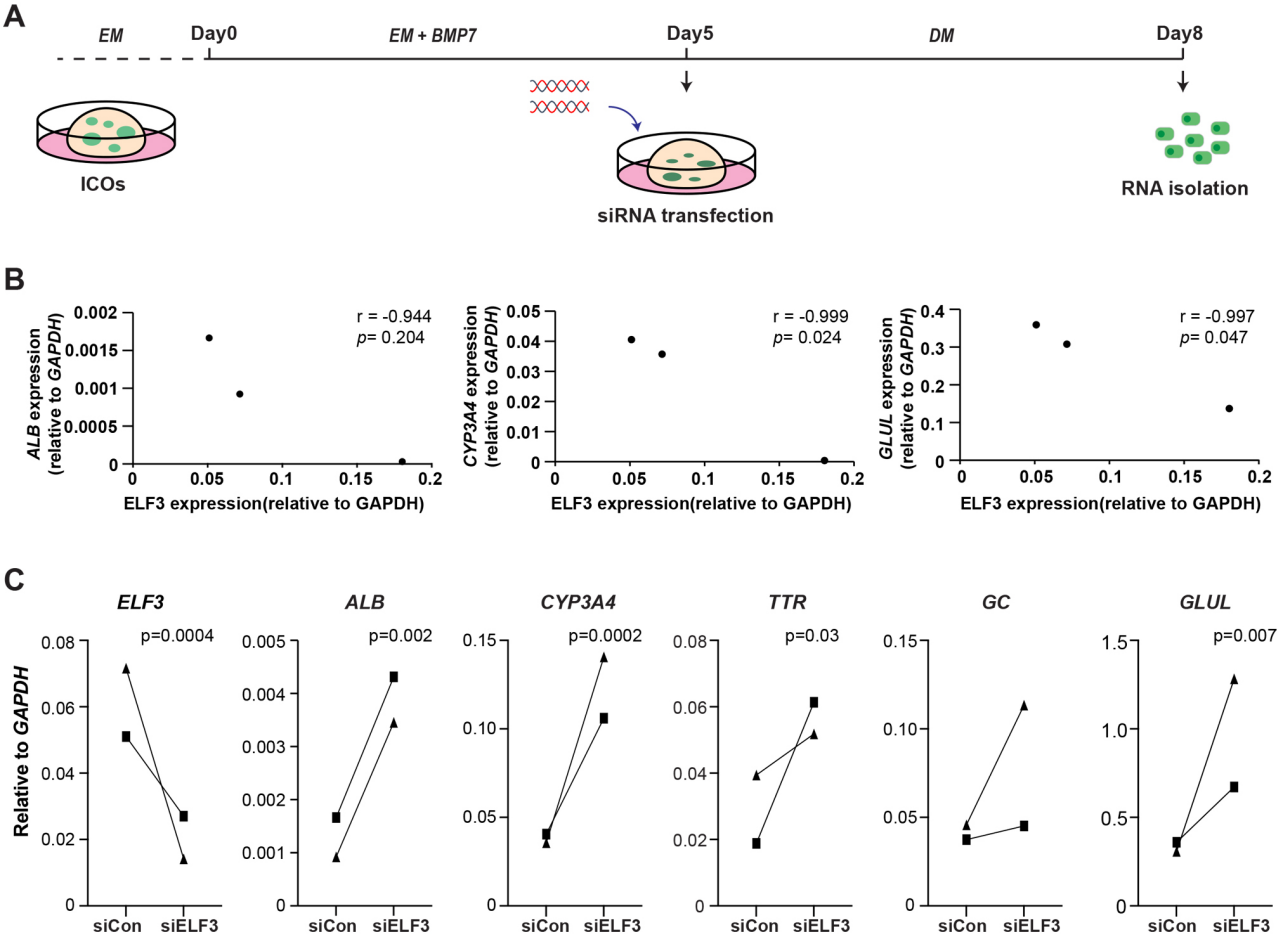
***ELF3* depletion promotes hepatic differentiation in ICOs *in vitro*.** (A) Experimental setup of siRNA transfection during hepatic differentiation of ICOs. ICOs were maintained in EM. EM-ICOs were first cultured in EM plus BMP7 for 5 days. On Day 5 during the differentiation, ICOs were transfected with siRNA [siCon (control siRNAs) and siELF3] and switched to DM until Day 8 (Day 3 post-transfection). Cells were harvested for RNA isolation for quantitative PCR. (B) Reverse transcription quantitative PCR (RT-qPCR) results comparing the expression of *ELF3* with the expression of hepatocyte markers *ALB*, *CYP3A4* and *GLUL* in three different ICO clones after differentiation. Correlation (*r*) and *P*-value were calculated using Pearson correlation analysis. (C) RT-qPCR results showing the expression levels of *ELF3* as well as those of hepatocyte markers *ALB*, *CYP3A4*, *TTR*, *GC* and *GLUL* in *ELF3*-targeting or non-targeting control siRNA-transfected DM-ICOs. Paired two-tailed Student’s *t*-test *P*-values are indicated above the graphs. *GAPDH* was used for normalization.

Next, we focused on ELF3, as this TF is highly expressed in ICOs compared to in PHHs. Also, the binding motif of ELF3 showed the strongest differential enrichment between open chromatin regions of DM-ICOs and PHHs. To investigate the effect of *ELF3* depletion during ICO differentiation, we performed *ELF3* knockdown using a combination of two siRNAs in three different ICO lines at Day 5 after differentiation (in DM), evaluating the expression of known hepatocyte markers (Fig. 4A). Interestingly, we already observed a strong negative correlation between expression of *ELF3* and expression of *ALB*, *CYP3A4* and *GLUL* within the three ICO clones even without *ELF3* knockdown (Fig. 4B). In two of the three clones, we observed a robust reduction (∼60% knockdown efficiency on average) in *ELF3* levels in the siRNA-transfected samples compared to the non-targeting siRNA control at differentiation Day 8 (day 3 post-transfection) (Fig. 4C). Next, we evaluated the expression of known hepatocyte markers by reverse transcription quantitative PCR (RT-qPCR) and observed increased levels of *ALB*, *CYP3A4*, *TTR*, *GC* and *GLUL* upon *ELF3* knockdown in both ICO lines tested (Fig. 4C). To further validate these findings, we repeated the *ELF3* knockdown and performed RNA-seq on transfected cells at both Day 8 (day 3 post-transfection) and Day 12 (day7 post-transfection). As expected, we detected upregulation of hepatocyte-associated genes in si*ELF3* knockdown samples compared to the control (siCon; control siRNAs) at both Day 8 and Day 12 (Fig. S5D). Interestingly, most cholangiocyte and liver progenitor markers remained largely unchanged, indicating that *ELF3* knockdown only is not sufficient to suppress the biliary epithelial feature in differentiated ICOs (Fig. S5D). Overall, our data indicate that lower *ELF3* levels associate with more mature hepatic-like features in ICOs under differentiation conditions, suggesting that ELF3 functions as a barrier of hepatic differentiation of ICOs *in vitro*.

## DISCUSSION

In this study, we applied an optimized liver tissue dissociation protocol to exclusively isolate primary hepatocytes from human liver. Currently available scRNA-seq datasets were mainly obtained from the whole liver to capture all cell types in the tissue (MacParland et al., 2018; Aizarani et al., 2019). However, this method usually leads to a limited and inconsistent proportion of PHHs from each donor, causing batch effects in the downstream computational analysis. We modified the two-step perfusion protocol to generate a pure hepatocyte population (98%) from liver tissue. Using scRNA-seq, we confirmed the known zonal heterogeneity within the isolated hepatocytes. Considering that some liver diseases, as well as infections, have shown zonal preference (Caldwell and Argo, 2010; Zhuang et al., 2020; Manco and Itzkovitz, 2021; Yang et al., 2021), we expect that enriched PHHs (potentially further sorted based on zonal cell surface markers, for which SLCO1B3 could be a candidate) would serve as a better and more precise resource for pericentral liver disease modeling. Laser capture microdissection (LCM) has been used to isolate human hepatocytes from different zones for comprehensive comparison of transcriptome and DNA methylome genome wide (Brosch et al., 2018). However, LCM extracts hepatocytes and non-parenchymal cells, and cryosectioning of the cells limits the usage for a broader downstream analysis.

The emergence of liver organoids has offered an alternative *in vitro* long-term culture system to better recapitulate liver tissue in a dish. However, organoid-derived hepatic-like cells are functionally limited compared to human hepatocytes, and the underlying biological mechanisms to promote differentiation remain unclear. Therefore, in this study, we investigated transcriptomic and epigenomic diversity between PHHs and ICOs. Integrative analysis of scRNA-seq, ATAC-seq and ChIP-seq datasets revealed that, besides in liver regeneration, AP-1 TFs could play a role in maintaining cellular functionality of mature PHHs by co-occupying regulatory regions with the liver-specific TF HNF4A. Although functional investigations are limited owing to the lack of a proper model in humans, evidence from other studies could further support our findings. For example, a defect in hepatogenesis has been described in *c-Jun* (also known as *Jun*) knockout mice, as *c-Jun* null mouse embryonic stem cells failed to differentiate to hepatocyte in adult livers of chimeric mice (Hilberg et al., 1993; Eferl et al., 1999). In addition, a dysregulated level of JUN is associated with several liver diseases including hepatitis B virus infection, metabolic dysfunction-associated steatotic liver disease (MASLD) and tumorigenesis (Trierweiler et al., 2016; Schulien et al., 2019). A recent study developed a deep-learning based model to identify TF motifs at active enhancers in murine hepatocytes and found AP-1 motifs to be present in these regions, which further supports our observation (Bravo González-Blas et al., 2024). These results all suggest that proper expression of JUN/AP-1 is essential in PHHs. AP-1 TFs including *JUN*, *FOS* and *ATF* are known to form homodimers/heterodimers and interact with other DNA-binding proteins (Shaulian and Karin, 2001). Our analysis showed that AP-1 TFs bind to the same regulatory elements as HNF4A in PHHs. Moreover, cooperation between AP-1 and ELF3, the key TF in ICOs, on transcription regulation has been described before in other cell types (Andreoli et al., 1997; Reddy et al., 2003; Kopp et al., 2007; Otero et al., 2012). Therefore, we reason that different interacting factors could explain the distinct binding profiles of AP-1 TFs in PHHs and ICOs. Alternatively, TF binding is cell-type specific, which is driven by the diverse chromatin states in different cells. The AP-1 motifs enriched in ICO-specific open chromatin regions indicate that AP-1 TFs could have variable regulatory targets and function differently in ICOs than in PHHs. Indeed, JUN and JUNB have been associated with the WNT signaling pathway (Mann et al., 1999; Eferl and Wagner, 2003; Nateri et al., 2005), which is highly active in ICOs. Therefore, we hypothesize that AP-1 TFs may function in supporting cell survival and proliferation because of an active WNT pathway in ICOs.

Motif analysis revealed high enrichment of PROX1 and SOX17 motifs in ICOs, consistent with the important roles of SOX factors in controlling the development of bile duct and cholangiocyte differentiation (Poncy et al., 2015; Merino-Azpitarte et al., 2017). However, the function of PROX1 in cholangiocytes or ICOs is less studied. Previous work has proposed PROX1 as an early marker for the developing liver required for the migration and differentiation of hepatoblasts (Burke and Oliver, 2002). Liver-specific inactivation of PROX1 leads to hepatic injury with very defective hepatocyte morphogenesis (Seth et al., 2014; Goto et al., 2017). Because PROX1 is highly expressed in hepatocytes and ICOs (human liver atlas data) (Aizarani et al., 2019), with potentially different occupancy preferences, our study has indicated a diverse regulatory function of PROX1 in PHHs and ICOs that requires further functional validation.

The TF ELF3 is highly expressed in biliary epithelial cells (MacParland et al., 2018; Suzuki et al., 2021). As reported, our gene regulatory comparison between PHHs and ICOs shows ELF3 to be exclusively active in ICOs, which display cholangiocyte-like features. Our functional study further reveals upregulation of hepatocyte markers upon *ELF3* depletion in ICOs, suggesting that ELF3 may function as a barrier in hepatocyte differentiation. To date, several studies have reported a role for ELF3 in regulating mesenchymal-to-epithelial/epithelial-to-mesenchymal transitions (MET/EMT), key biological processes that occur in many physiological events, such as development, embryogenesis and cell reprogramming (Yeung et al., 2017; Liu et al., 2019; Sengez et al., 2019). In the liver, MET/EMT dynamics are associated with liver progenitor cell identity and hepatocyte (de)differentiation upon *in vitro* culture (Cicchini et al., 2015; Xiang et al., 2019; Katsuda et al., 2020). Thus, the loss of ELF3 may affect the expression of a set of hepatocyte genes through MET/EMT processes. Next to perturbation of ELF3, introducing TFs and co-factors necessary for PHHs, such as MLX and PPARGC1A, into ICOs would offer an alternative strategy for the improvement of functional hepatocyte generation from ICOs, which has been successfully described before in other *in vitro* models (Velazquez et al., 2021).

In conclusion, our study describes a comprehensive comparison of gene regulatory mechanisms between PHHs and ICOs. These findings provide new biological insights into PHHs as well as ICOs, which will benefit the development of functional human hepatocyte models *in vitro*.

## MATERIALS AND METHODS

### Ethics statement

Primary human liver cells were freshly isolated from patients undergoing liver surgery. General approval for use of remnant, anonymized surgical material was granted in accordance with the Dutch ethical legislation as described in the Medical Research (Human Subjects) Act and confirmed by the Committee on Research involving Human Subjects, in the region of Arnhem-Nijmegen, the Netherlands (CMO-2019-5908). Liver samples were generated from five cancer patients of different ages and sexes who underwent liver resections, and only the healthy resection margins of the liver tissue were collected for the use in this study. Notably, although this part of the liver is free of detectable tumors, we cannot exclude the possibility that it might have been affected by the presence of the tumor or the treatment the patient received. Metadata associated with these samples can be found in Table S3. Collection of liver resection margins was conducted according to the principles expressed in the Declaration of Helsinki. The use of liver samples for research was approved by the Medical Ethical Committee (MEC) of the Erasmus University Medical Center (Erasmus MC) (MEC-2014-060) and by MEC-Radboud University Medical Center (CMO-2015-2062).

### Human liver tissue dissociation and single-cell isolation

Human liver specimens were immediately stored at 4°C in Dulbecco's modified Eagle medium (DMEM)/F12 for less than 1 h, after which they were used for primary hepatocyte isolation using the two-step perfusion protocol described previously (Yang et al., 2021). Surgical resection margins of the liver obtained for this study weighed minimally 10 g. Briefly, the liver segment was first perfused with HBSS (Thermo Fisher Scientific, 14170-138) containing 0.64 mM EDTA (Thermo Fisher Scientific, 15575-038) and 10 mM HEPES (Thermo Fisher Scientific, 15630-056) to wash away the blood, and then perfused with HBSS supplemented with 10 mM HEPES to remove any residual EDTA. We did not make a distinction between veins/arteries used. Next, the liver segment was perfused with HBSS supplemented with 10 mM HEPES, 0.75 mg/ml CaCl_2_ and low-concentration collagenase VIII (3333 units per 50 ml), followed by perfusion using the same buffer but with high-concentration collagenase VIII (13,333 units per 50 ml) for ∼30 min until the liver tissue became soft. All the perfusion steps were performed at 37°C. After collagenase dissociation, DMEM (Thermo Fisher Scientific, 31885-023) with 10% fetal bovine serum was used to inactivate collagenase activity, and liver tissue was cut into small pieces to release cells into the DMEM. The resulting cell suspension was collected and filtered with a 100 µm cell strainer. The cells were pelleted at 50 ***g*** for 5 min. To remove non-hepatocytes, cells were washed with DMEM and pelleted for 5 min at 50 ***g*** two more times (until the supernatant was clear after the centrifugation). To remove the non-viable hepatocytes, the cell pellet was resuspended in 28% Percoll and centrifuged at 150 ***g*** for 20 min. Cells were counted with Trypan Blue, and Percoll gradient centrifugation was repeated until the percentage of dead cells was ∼20%. The yield of hepatocytes ranged anywhere between 20 million and 200 million. Their viability following Percoll density gradient purification reached ∼80-90%. The isolated hepatocytes were directly used for single-cell sorting.

### ICO culture, differentiation and single-cell isolation

ICOs (*n*=3) were obtained from Erasmus MC and from the biobank at the Department of Gastroenterology and Hepatology, Radboud University Medical Center (Nijmegen, The Netherlands). To initiate ICOs, tissue biopsies from liver (∼0.5-3 cm^2^, *n*=1) were obtained from donor livers during liver transplant procedures performed at the Erasmus MC (Rotterdam, The Netherlands). ICOs were initiated, cultured and differentiated towards hepatocyte-like cells as published before (Huch et al., 2015; Broutier et al., 2016). Briefly, single cells were dissociated from liver biopsies, and ∼10,000 cells were seeded with basement membrane extract (BME; Trevigen, 3533-010-02). After the BME became solid, the initiation medium was added. Following the establishment of the culture, organoids were maintained in EM every 3 days. For the passaging, organoids were released from old BME, mechanically dissociated into small pieces and reseeded into new BME. Passaging was performed in a 1:5 splitting ratio once every 7-10 days, and all the organoids used in this study were within ten passages. For hepatocyte differentiation, the organoids were first cultured in EM supplemented with BMP7 to accelerate the proliferation of hepatocytes (PEPROTECH, 120-03P) for 5 days, and then switched to DM for more than 10 days (Sugimoto et al., 2007). To obtain single cells, BME was removed by tenfold dilution in cold DMEM/F-12 (Thermo Fisher Scientific, 12634-010). The organoids were then digested using 0.05% Trypsin-EDTA (Thermo Fisher Scientific, 25300-054) until 80-90% of the cells were single cells. Trypsin was removed, and cells were collected for further use. Components of the initiation medium, EM and DM are listed below.

#### Initiation medium

Advanced DMEM (AdDMEM)/F12 (Thermo Fisher Scientific) supplemented with Hepes (Thermo Fisher Scientific), Glutamax (Thermo Fisher Scientific), 1% N2 (Thermo Fisher Scientific), 1% B27 (Thermo Fisher Scientific), 1.25 mM N-acetylcysteine (Sigma-Aldrich), 10 nM gastrin (Sigma-Aldrich), 10 mM nicotinamide (Sigma-Aldrich), 50 ng/ml hEGF (Peprotech), 10% RSPO1 conditioned medium (homemade), 100 ng/ml FGF10 (Peprotech), 25 ng/ml HGF (Peprotech), 5 μM A83.01 (Sigma-Aldrich), 10 μM FSK (R&D Systems), 25 ng/ml Noggin (Peprotech), 30% Wnt-3A conditioned medium (homemade) and 10 μM Y27632 (Selleck).

#### EM

AdDMEM/F12 (Thermo Fisher Scientific) supplemented with Hepes (Thermo Fisher Scientific), Glutamax (Thermo Fisher Scientific), 1% N2 (Thermo Fisher Scientific), 1% B27 (Thermo Fisher Scientific), 1.25 mM N-acetylcysteine (Sigma-Aldrich), 10 nM gastrin (Sigma-Aldrich), 10 mM nicotinamide (Sigma-Aldrich), 50 ng/ml hEGF (Peprotech), 10% RSPO1 conditioned medium (homemade), 100 ng/ml FGF10 (Peprotech), 25 ng/ml HGF (Peprotech), 5 μM A83.01 (Sigma-Aldrich) and 10 μM FSK (R&D Systems).

#### DM

AdDMEM/F12 medium supplemented with Hepes (Thermo Fisher Scientific), Glutamax (Thermo Fisher Scientific), 1% N2 (Thermo Fisher Scientific), 1% B27 (Thermo Fisher Scientific), 1 mM N-acetylcysteine (Sigma-Aldrich), 10 nM gastrin (Sigma-Aldrich), 50 ng/ml hEGF (Peprotech), 10 nM gastrin (Sigma-Aldrich), 50 ng/ml hEGF (Peprotech), 25 ng/ml HGF (Peprotech), 100 ng/ml FGF19 (Peprotech), 25 ng/ml BMP-7 (Peprotech), 0.5 μM A83.01 (Sigma-Aldrich), 10 μM DAPT (Sigma-Aldrich) and 30 μM dexamethasone (Sigma-Aldrich, D4902).

### Immunofluorescence for organoids

BME was first removed using Cultrex Organoid Harvesting Solution (Trevigen, 3700-100-01), and ICOs were collected. Cells were fixed with 4% paraformaldehyde on ice for 30 min and then washed three times with cold PBS. After fixation, cells were blocking using blocking buffer [0.5% Triton-1000, 1% bovine serum albumin (BSA), 1% dimethyl sulfoxide in PBS)] at room temperature for 3 h. Then, primary antibodies [anti-EPCAM (eBioscience, 14-9326-82; 1:100) and anti-ALB (Bethyl Laboratories, A80-229A; 1:50)] were diluted and incubated with cells at 4°C for more than 24 h. After three times washing with PBS+1% BSA, cells were incubated with secondary antibodies [donkey anti-goat Alexa Fluor 594 (Thermo Fisher Scientific, A32758; 1:250) and donkey anti-goat Alexa Fluor 488 (Thermo Fisher Scientific, A-21202; 1:250)] for 2 h at room temperature. Cells were washed three times with PBS+1% BSA and stained with Hoechst 33342 (Thermo Fisher Scientific, 62249) for 1 h at room temperature. Finally, cells were mounted in the chamber with the mounting buffer (IBIDI, 50001) and imaged by a Leica SP8 confocal microscope. Images were further processed using ImageJ (Schneider et al., 2012).

### siRNA transfection

At Day 5 of ICO differentiation, cells were collected. For transfection, siRNAs (negative control No.1 siRNA, Thermo Fisher Scientific, 4390843; siELF3-s4623 and siELF3-s4624, Thermo Fisher Scientific, 4427037) with a final concentration of 40 nM and Lipofectamine 3000 (Thermo Fisher Scientific, L3000015) reagent were used following the manufacturer's protocol (Broutier et al., 2016). To obtain efficient knockdown, two separate siRNAs targeting *ELF3* were combined. After transfection, cells were seeded in BME and cultured in DM supplemented with 10 µM Rock inhibitor (Gentaur, A3008) for the first 3 days. Transfected cells were then harvested for RNA isolation at Day 8 and Day 12 after differentiation.

### RNA extraction and RT-qPCR

Cells collected from 3D culture organoids or sorted cells were lysed in RLT buffer from an RNeasy kit (Qiagen, 74106). RNA samples were isolated using the same kit following the manufacturer's recommendations. 100-300 ng RNA was used for cDNA synthesis using an iScript cDNA Synthesis Kit (Bio-Rad, 1708891) following the manufacturer's manual. RT-qPCR was performed in triplicate on a CFX96 Real-Time System (Bio-Rad) with SYBR Green Supermix (Bio-Rad, 1725006CUST). Data were normalized to *GAPDH*. Primer sequences are provided in Table S4.

### Bulk RNA-seq library preparation

For library construction, 100-250 ng total RNA was used with a KAPA RNA Hyper+RiboErase HMR Kit (Roche, 08098140702), following the manual. After library construction, libraries were quantified on a bio-analyzer using a DNA high-sensitivity kit (Agilent, 5067-4626) and sequenced paired-end (2×38 bp) on an Illumina NextSeq 500 platform.

### ATAC-seq library preparation

Cells (25,000) were washed once with cold PBS and then resuspended in Tn5 reaction mix [12.5 µl 2× TD buffer (homemade), 1.25 µl Tn5 enzyme (homemade), 0.5 µl Digitonin (Promega, G9441) and 11 µl H_2_O]. Cells were incubated in a thermo mixer at 37°C, 800 rpm for 20 min. After the incubation, reaction cleanup was performed using a Qiagen MinElute Reaction Cleanup Kit (28204). The purified tagmented DNA samples were barcoded with Nextera Index Kit primers and amplified via PCR followed by AMpureXP bead (Agencourt, A63882) size selection. Briefly, after the first five PCR cycles, 5 µl PCR reaction was used for quantitative PCR (qPCR) to determine the number of additional cycles needed. After amplification, the PCR reaction was cleaned up with 0.65× AMpureXP beads followed by 1.8× SPRI beads to select DNA fragments from 150 bp to 600 bp. Finally, libraries were quantified on the bio-analyzer using a DNA high-sensitivity kit (Agilent, 5067-4626), and sequenced on an Illumina NextSeq 500 with 38 bp paired-end reads at a depth of ∼20 million reads per sample.

### Single-cell cDNA library preparation

Single-cell RNA libraries were constructed according to the mCEL-seq2 protocol (Hashimshony et al., 2016). Briefly, 100 nl lysis buffer [0.2% Triton X-100, 2 mM dNTPs, 1:50.000 ERCC ExFold RNA spike-in (Thermo Fisher Scientific, 4456739)] was added per well using a Nanodrop Ns-2 Stage (BioNex). For the *in vitro* reverse transcription reaction, SuperScript™ II Reverse Transcriptase (Invitrogen, 18064-14) was used with the following the protocol: 4°C for 5 min; 25°C for 10 min; 42°C for 60 min; 70°C for 10 min. Next, 960 nl second-strand reaction mix containing *E. coli* DNA ligase (NEB, M0205L), *E. coli* DNA polymerase I (NEB, M0209L) and random hexamer (Sigma-Aldrich, 11034731001) were added per well for second-strand synthesis following the protocol 16°C for 2 h. All the cDNA from one 384-well plate was pooled. *In vitro* transcription was performed using a MEGAscript™ T7 Transcription Kit (Invitrogen, AMB 1334-5) by incubating the reaction at 37°C for 14 h. The amplified RNA was fragmented and used for cDNA synthesis using Superscript II and the random octamer primer. Libraries were amplified for nine cycles for the hepatocyte libraries and eight cycles for the liver organoid libraries using Phusion^®^ High-Fidelity PCR Master Mix (NEB, M0531S) with the Nextflex primers, and quantified on the bio-analyzer using a DNA high-sensitivity kit (Agilent, 5067-4626). Barcoded libraries were sequenced on an Illumina NextSeq 500 with 38 bp paired-end reads at a depth of ∼25 million reads per plate.

### scRNA-seq data analysis

Barcode information was extracted from read 1 and added to the ID of paired read 2 using UMI-tools 1.1.0 (Smith et al., 2017). The barcoded reads containing UMIs (1-8 bp) and cell barcode (9-16 bp) were not used for mapping. Only the paired read 2 were aligned to a modified human genome (hg38) containing 92 ERCC spike-in sequences using STAR-2.7.6a with default settings (Dobin et al., 2012). After mapping, featureCount 2.0.1 was used to assign the reads to genes (Liao et al., 2014). The number of UMIs per gene was counted for each cell and the gene–cell count tables were generated using UMI-tools 1.1.0 with the following parameters: --per-gene, --per-cell. For the downstream analysis, Seurat 3.2.3 was applied for data filtering, batch correction, normalization, clustering, visualization and differentially expressed gene analysis (Stuart et al., 2019). Briefly, Seurat objects were created per donor using gene–cell matrices. Genes detected (UMIs>0) in at least five cells were kept. Cells with less than 1000 UMIs or more than 50 mt% were removed. UMI counts were then log2 transformed and normalized to sequencing depth per cell, and number of total UMIs and mt% were regressed out during the scaling to correct for sequencing depth and mt%.

For scRNA-seq data analysis within PHHs or ICOs, to minimize the batch effect, Seurat objects from different donors were integrated using the functions FindIntegrationAnchors and IntegrateData. Next, the top 2000 variable genes were found and used for principal component analysis (PCA), with the setting npcs=50. Seurat functions FindNeighbors, FindClusters and RunUMAP were used to construct a Shared Nearest Neighbor (SNN) Graph, identify clusters of cells based on the SNN graph, and finally reduce the dimension for visualization. Finally, differentially expressed genes from each cluster were identified using Wilcoxon rank sum test, and the significant ones [adjusted *P* (adj-*P*)<0.05] were used for pathway analysis via Enrichr web tool (Kuleshov et al., 2016; Xie et al., 2021).

For integration analysis of PHHs and ICOs, PHH and ICO Seurat objects were merged using Seurat merge function. To compare ICOs to PHHs only, the non-hepatocyte cluster in the PHHs dataset was not included in the integration. The newly merged Seurat object was first scaled by regressing out the number of total UMIs, mt% and biological replicates, and then used for further analysis following the same procedure as mentioned before.

### Correlation analysis with zonally expressed human and mouse transcripts

For correlation analysis of zonally expressed genes between human and mouse hepatocyte, we used a set of 94 genes previously identified to be expressed in both human and mouse (Halpern et al., 2017; MacParland et al., 2018). Average expression levels of each gene were calculated for all the four hepatocyte clusters (from this study) and then scaled and centered by *z*-scores. The same analysis was applied for nine layers of mouse hepatocytes (Halpern et al., 2017). Pearson correlation was calculated to compare the four human hepatocyte clusters with the nine layers of mouse hepatocytes.

### Cell type prediction analysis of ICOs

To predict the cell type of the cells in ICOs, we used two published scRNA-seq datasets generated from the human liver as references (MacParland et al., 2018; Andrews et al., 2022), extracted the data of hepatocytes from all zones and cholangiocytes, and trained a cell type Random Forest classifier by Python package sklearn (v.0.24.2). Only the top 5000 variable genes were using for the training. Next, ICOs from both culture conditions were classified using this classifier and assigned to a cell type in the reference for each single cell. Finally, Sankey plots were generated based on the assignments using R package networkD3 (v.0.4).

### TF regulon analysis

SCENIC R version (Aibar et al., 2018) was used for gene regulatory network analysis. Briefly, raw gene–cell count matrix was extracted from ‘RNA’ assay in Seurat object and used as input for SCENIC. The matrix was log2 transformed, and co-expression network was calculated using runGenie3 with default setting. To predict the TF regulons, *hg19-tss-centered-10kb-7species.mc9nr.feather* motif database from RcisTarget was used. Regulons were created with the following parameters, minGenes=20, coexMethod=“top5perTarget”. The area under the recovery curve (AUC) scores were generated per cells and imported into Seurat for downstream analysis and visualization.

### Bulk RNA-seq analysis

Paired-end reads were aligned to the human genome (hg38) using STAR-2.7.6a (Dobin et al., 2012) with default settings. To have comparable sequencing depth from each sample, subsampling was applied to the datasets according to the amount of reads from the sample with lowest sequencing depth using SAMtools 1.7 (Li et al., 2009). The number of mapped fragments was quantified at gene level using featureCount 2.0.1 (Liao et al., 2014) with the following parameters, -p -g gene_name, based on Gencode annotation (v30). The R package DEseq2 1.26.0 (Love et al., 2014) was used for differential gene expression analysis. Genes with less than two fragments were removed from the differential gene expression analysis. All the differentially expressed genes were determined according to adj-*P*<0.05. For gene set enrichment analysis, fgsea package from R Bioconductor was used following the online tutorial. Datasets from a previous study were used for the comparisons (Brosch et al., 2018). An enrichment score normalized to mean enrichment of random samples with the same size was calculated, and adj-*P*<0.05 generated from Benjamini–Hochberg method was applied as a threshold of significance.

### ATAC-seq and ChIP-seq analysis

For ATAC-seq data analysis, pair-end reads were mapped to the human reference genome (hg38) using BWA 0.7.17-r1188 mem (Li and Durbin, 2009) with default settings. Next, duplicates and reads with mapping quality lower than 30 were removed using samtools 1.7 (Li et al., 2009). Open chromatin profile tracks were generated using deeptools 3.5.0 (Ramírez et al., 2016) bamCoverage function with the following setting: --normalizeUsing CPM. For peak calling, MACS2 v2.2.7.1 (Zhang et al., 2008) was used with the following settings: -g hs, --nomodel. Overlapping and unique peak sets between biological replicates or different samples were identified using homer v4.11.1 (Heinz et al., 2010) mergePeaks function with the parameter -d given. To minimize the donor variation and get most confident peak sets, only the peaks presented from both biological replicates were used for further analysis. For Spearman correlation analysis, ATAC peaks were first merged from all the samples, and the ATAC signal was quantified in all peaks from each sample using bedtools v2.29.2 multicov function (Quinlan and Hall, 2010). The peak-sample count table was either imported into DEseq2 1.26.0 (Love et al., 2014) for making the PCA plot following the online tutorial, or count per million (CPM) normalized for Spearman correlation analysis using rcorr function from R package Hmisc 4.4-2. Genes targeted by HNF4A, JUND or both proteins were selected according to the following criteria: expression level higher than 100 UMIs from all summed single cells and the binding sites detected within TSS±5 kb of the genes. Next, HNF4A- and JUND-targeted genes were used for GO analysis using Enrichr web tool. For motif analysis, GimmeMotifs v0.15.1 (van Heeringen and Veenstra, 2011) was applied using 200 bp around peak summit regions as input with the following settings: -s 0, -b gc. For differentially enriched motif analysis, the unique peak sets from each sample were merged. Then, normalized read coverage was quantified (as CPM) in each peak region (200 bp around summit point) for each sample. After log2 transformation, the matrixes were imported into GimmeMotifs (van Heeringen and Veenstra, 2011), and maelstrom function was used with the default settings. In case multiple TFs were predicted to bind to the enriched motifs, we checked their expression levels and only listed the factors that were clearly detected in the scRNA-seq data.

For ChIP-seq analysis, the ChIP-seq profiles were downloaded from ENCODE (ENCODE Project Consortium, 2012; Ramaker et al., 2017; Davis et al., 2018). Read mapping, filtering and peak track generation were done using the same strategy as for ATAC-seq analysis. MACS2 was used for peak calling with the following settings: -g hs. For Spearman correlation analysis, ChIP-seq signal was quantified for each TF in the ATAC peak regions in PHHs. After CPM normalization, the count table was imported into R, and correlation was calculated using the same strategy as ATAC-seq Spearman correlation analysis. The intersecting peak regions were identified using homer mergePeaks with the parameter -d given. For the motif analysis, GimmeMotifs was applied with the settings as mentioned before (van Heeringen and Veenstra, 2011). Details of ChIP-seq data from ENCODE used in this study can be found in Table S5.

### Quantification and statistical analysis

For studies in which statistical analyses were performed, at least three biological replicates were used, unless otherwise indicated. For two-condition comparisons, paired two-tailed Student’s *t*-tests were used with *P*<0.05 as the threshold for significance. For multiple testing, a Wilcoxon rank sum test was used in Seurat (as default) and a Wald significance test was used in DEseq2 (as default) for differentially expressed gene determination, with adj-*P*<0.05 as the threshold for significance. For pathway and gene set enrichment analysis, Fisher exact test and Benjamini–Hochberg method were used as suggested with adj-*P*<0.05 as the threshold for significance.

## Supplementary Material

10.1242/dmm.050883_sup1 Supplementary information

Table S1. scRNA differentially expressed genes in each cluster
