# Supplementary material for: Multi-omics analysis reveals distinct gene regulatory mechanisms between primary and organoid-derived human hepatocytes
Source: Dis Model Mech. 2025 Jan 29;18(1):dmm050883. doi: 10.1242/dmm.050883 (PMC11810045; doi:10.1242/dmm.050883)
Supplement: Supplementary information [file dmm-18-050883-s1.pdf]

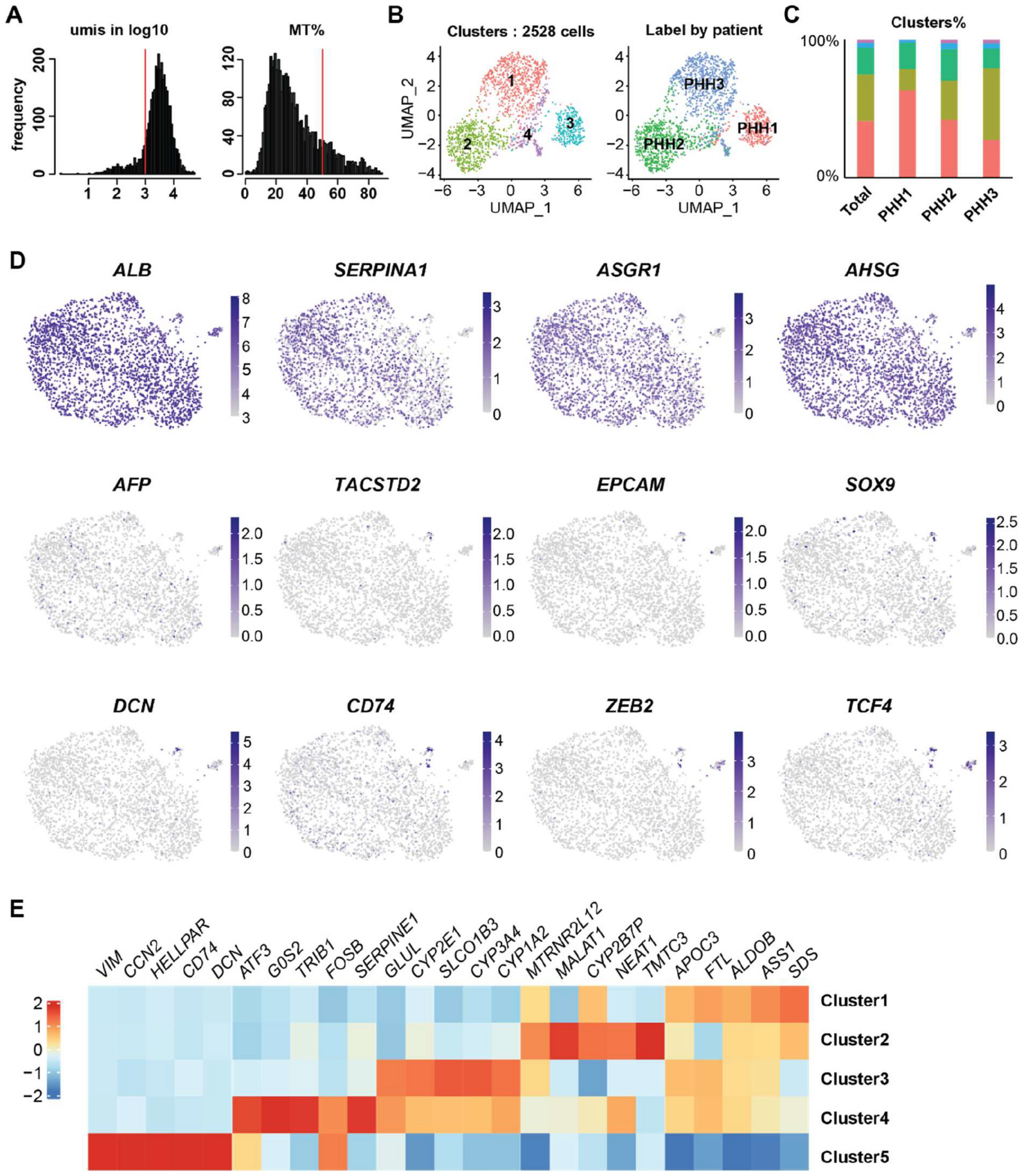

Fig S1. scRNA-seq analysis characterizing liver cells isolated using an optimized two-step perfusion protocol shows nearly pure hepatocyte population.

(**A**) Histograms showing the numbers of UMIs (in log10 format) in each cell on the left, and percentage of mitochondrial gene expression per cells on the right. Only the cells following the

Disease Models & Mechanisms • Supplementary information

criteria (1000 < UMIs, MT% < 50%) are used for the downstream analysis. (**B**) UMAP plots highlighting the seurat clusters on the left and clusters labeled by donors on the right prior to batch correction. (**C**) Bar plot showing the fraction of cells contributed to each cluster by donors after batch correction. (**D**) UMAP plots of expression of mature hepatocyte markers (*ALB*, *SERPINA1*, *ASGR1* and *AHSG*), liver progenitor markers (*AFP* and *TACSTD2*), cholangiocyte markers (*EPCAM* and *SOX9*), and immune or epithelial cell types associated genes (*DCN*, *CD74*, *ZEB2*, and *TCF4*). Data was normalized to sequencing depth and shown in log2 format. (**E**) Heatmap showing the expression level of top 5 enriched genes from each cluster. Data is shown in Z-score format.

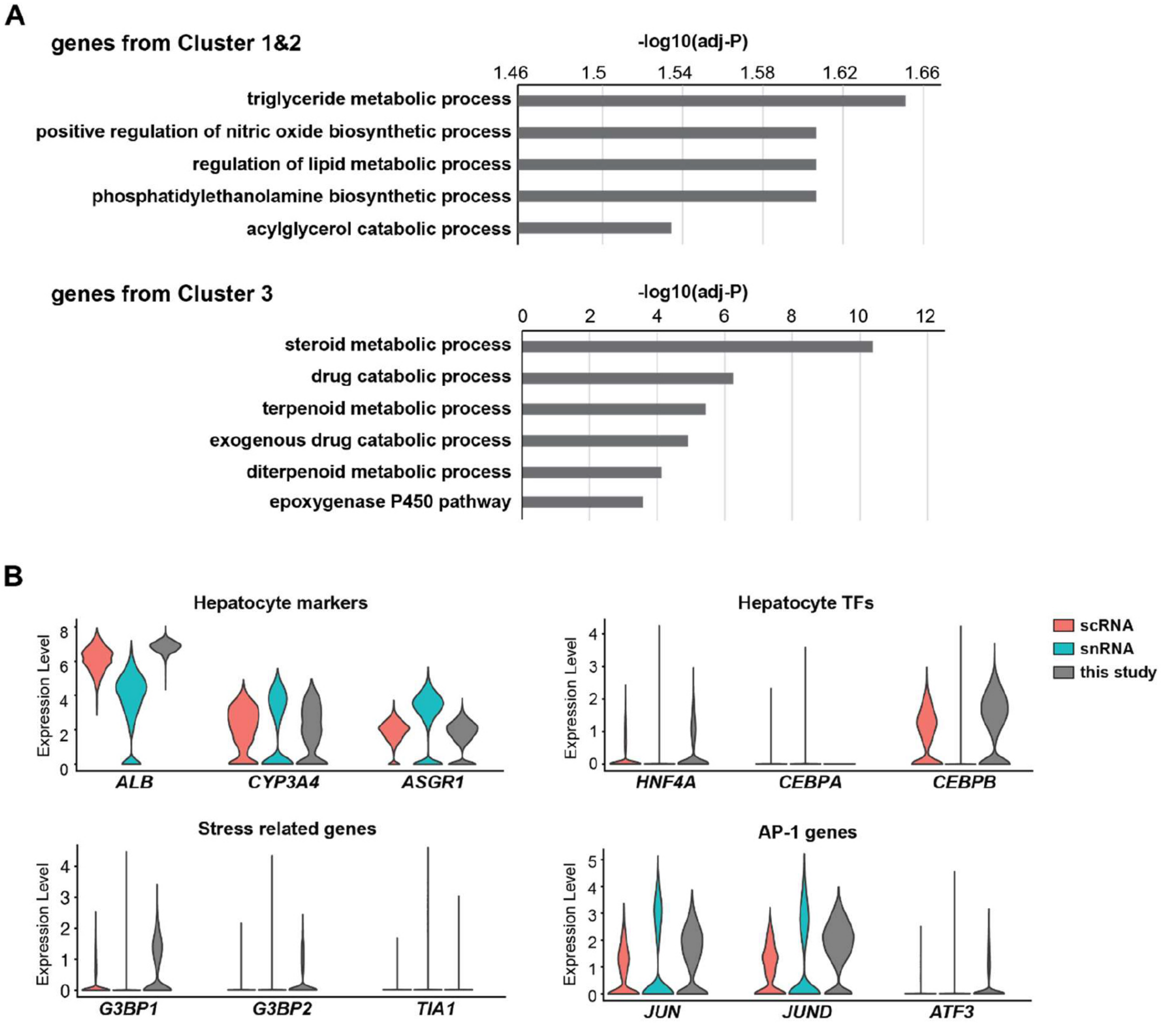

Fig S2. PHH clusters are enriched for typical hepatic processes

(**A**) GO analysis (biological process) for the typical gene sets from cluster 1, 2 and 3. Pathways were listed according to -log10 (adj-P value). (**B**) Violin plots showing the expression of selected hepatocyte markers and genes from AP-1 factors in single-cell/nuclei data from Brazovskaja et al., and this study. Data was normalized to sequencing depth and shown in log2 format.

Disease Models & Mechanisms • Supplementary information

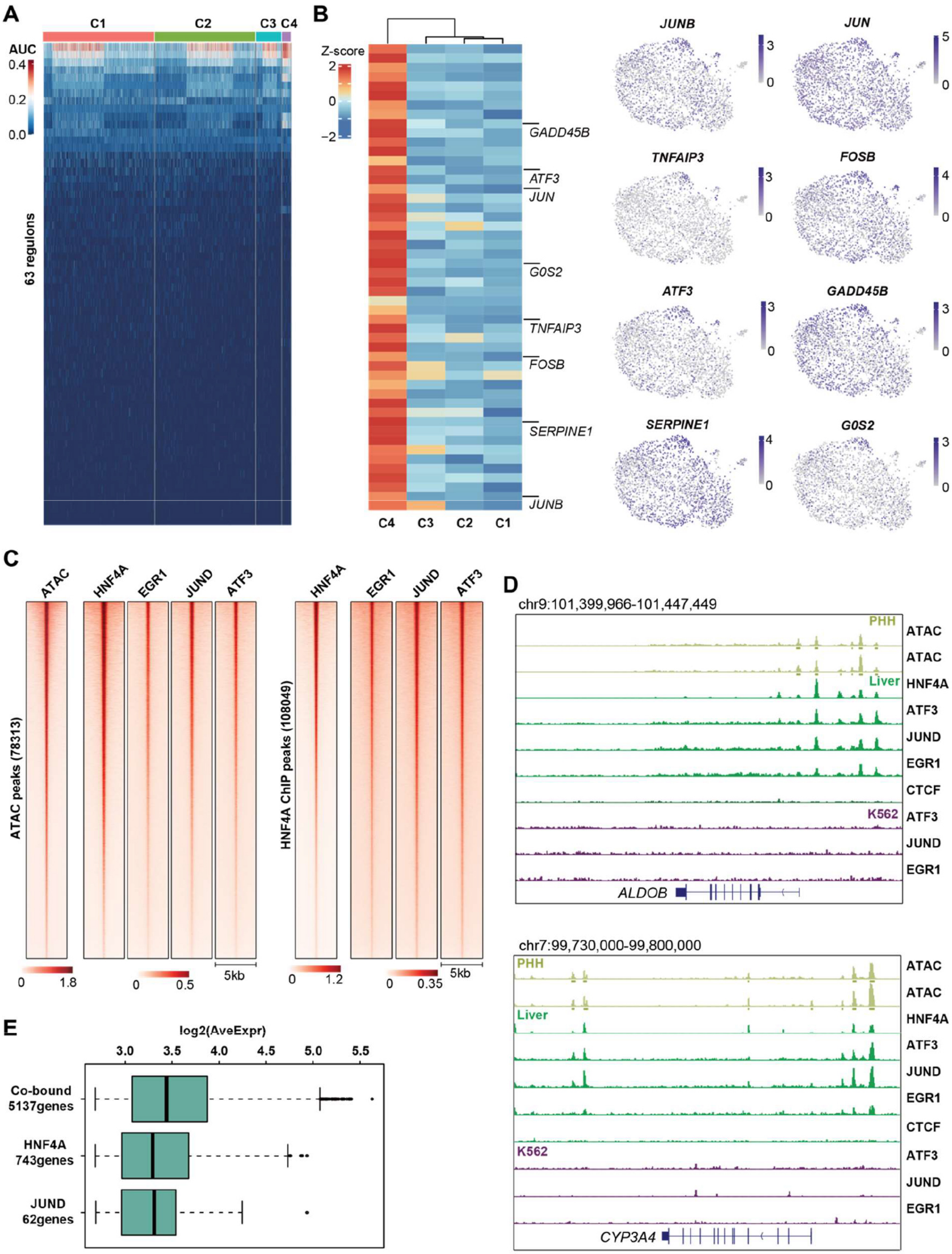

Disease Models & Mechanisms • Supplementary information

Fig S3. Integrative analysis shows the crosstalk between AP-1 TFs and HNF4A in PHHs.

(**A**) Heatmap of the AUC score of all 63 regulons analyzed by SCENIC, showing little difference between clusters. Score was shown in each single cells and cells were grouped in Seurat clusters. (**B**) Heatmap showing the expression of Cluster4 specific genes in z-score format. Genes associated to hepatocytes in the priming phase are highlighted on the right side (on the left) and UMAP plots displaying the expression levels of *JUNB*, *JUN*, *TNAIP3*, *FOSB*, *ATF3*, *GADD45B*, *SERPINE1* and *G0S2* (on the right). Data was normalized to sequencing depth and shown in log2 format. (**C**) Heatmap showing the enrichment of ATAC signal as well as HNF4A, EGR1, JUND, and ATF3 occupancies in 78313 ATAC peak regions (summit point ±2.5kb) in PHHs (on the left), and the enrichment of HNF4A, EGR1, JUND, and ATF3 occupancies in 108049 HNF4A binding sites (summit point ±2.5kb) in liver (on the right). Data was shown as CPM. (**D**) Genome browser screenshot showing the co-binding of HNF4A, ATF3, JUND, and EGR1 at *ALDOB* and *CYP3A4* genomic loci in liver, which overlaps with ATAC signal (identified peaks were indicated by underlying blocks) in PHHs. This co-localization was not observed from either CTCF in liver, or ATF3, JUND, EGR1 in K562 lymphoblast cell line. (**E**) Boxplot showing the average expression level of genes that are bound by both JUND and HNF4A (co-bound), HNF4A only and JUND only. Genes targeted by HNF4A, JUND or both proteins were selected according the following criteria, expression level higher than 100 umis from all summed single cells and the binding sites detected within TSS±5kb of the genes. Data was shown as log2 (total UMIs/ total number of cells in scRNA-seq *1000)

Disease Models & Mechanisms • Supplementary information

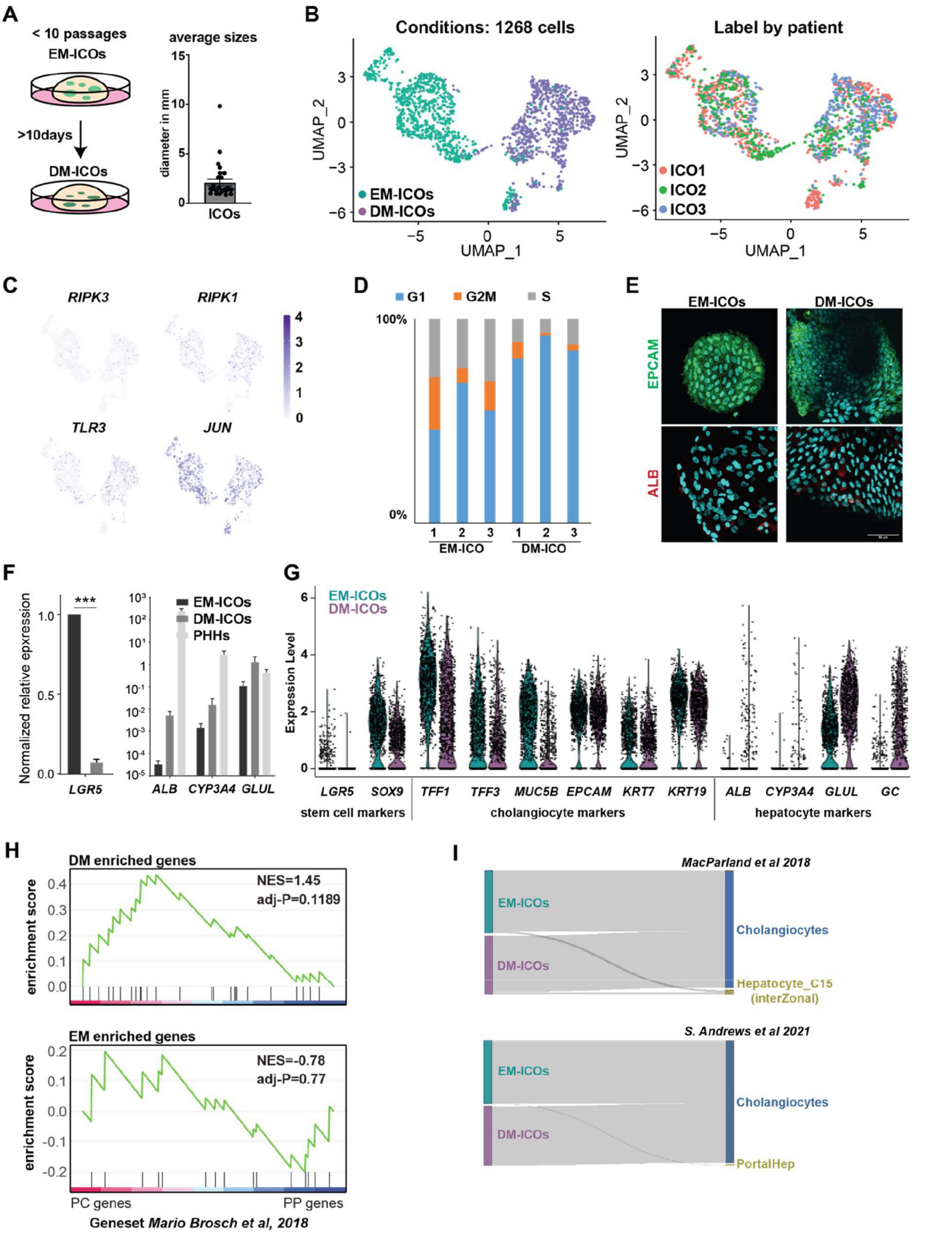
A
< 10 passages
EM-ICOs
>10days
DM-ICOs
average sizes
diameter in mm
ICOs
B
Conditions: 1268 cells
Label by patient
UMAP_1
UMAP_2
EM-ICOs
DM-ICOs
ICO1
ICO2
ICO3
C
RIPK3
RIPK1
TLR3
JUN
D
G1
G2M
S
100%
0%
EM-ICO
DM-ICO
E
EM-ICOs
DM-ICOs
EPCAM
ALB
F
Normalized relative epxression
***
LGR5
EM-ICOs
DM-ICOs
PHHs
ALB
CYP3A4
GLUL
G
EM-ICOs
DM-ICOs
Expression Level
LGR5
SOX9
TFF1
TFF3
MUC5B
EPCAM
KRT7
KRT19
ALB
CYP3A4
GLUL
GC
stem cell markers
cholangiocyte markers
hepatocyte markers
H
DM enriched genes
NES=1.45
adj-P=0.1189
enrichment score
EM enriched genes
NES=-0.78
adj-P=0.77
PC genes
PP genes
Geneset Mario Brosch et al, 2018
I
MacParland et al 2018
EM-ICOs
DM-ICOs
Cholangiocytes
Hepatocyte_C15 (interZonal)
S. Andrews et al 2021
EM-ICOs
DM-ICOs
Cholangiocytes
PortalHep

Fig S4. DM-ICOs display hepatocyte features compared to EM-ICOs.

(**A**) On the left, schematic overview of the ICO culture. ICOs were maintained in EM. For hepatic differentiation, ICOs were cultured in DM for more than 10 days. On the right, bar plot showing the average size of ICOs in diameter. All the experiments were performed using the ICO lines within 10 passages. (**B**) UMAP plots of single-cell transcriptome labeled by culture condition (left) or donors (right). Cells from different donors are separated mainly by the culture condition. (**C**) UMAP plots displaying the expression levels of *RIPK3*, *RIPK1*, *TLR3*, and *JUN*. Data was normalized to sequencing depth and shown in log2 format. **(D)** Bar plot showing the distribution of cells from EM-ICOs and DM-ICOs in different cell-cycle phases (G1, S, G2M). Cells were grouped based on the donors. (**E**) Immunostaining of EPCAM and ALB in EM-ICOs and DM-ICOs. Hoecht staining for the nuclei was used as a control. (**F**) qPCR results showing the expression levels of *LGR5*, *ALB*, *CYP3A4*, and *GLUL* in PHHs, EM-ICOs, and DM-ICOs. *GAPDH* was used as the house keeping gene. Expression was normalized to EM-ICO samples. Data was generated with 3 biological replicates. Mean ± SEM. Student t-test ****P<0.001*. (**G**) Violin plots showing the expression of selected genes such as WNT target gene *LGR5*, progenitor/cholangiocyte marker *SOX9*, mature cholangiocyte markers (*TFF1*, *TFF3*, *MUC5B*, *EPCAM*, *KRT7*, and *KRT19*), and mature hepatocyte markers (*ALB*, *CYP3A4*, *GLUL*, and *GC*). Cells were grouped based on culture conditions. (**H**) GSEA of DE genes between EM-ICOs and DM-ICOs compared to a zonal hepatic gene set obtained from a published study, showing that DM or EM genes were not enriched to any zonal expression profile. (**I**) Sankey plots showing the predicted cell types of ICOs in this study by Random Forest classifier based on transcriptomes of liver cells (hepatocytes and cholangiocytes) from two independent studies.

Disease Models & Mechanisms • Supplementary information

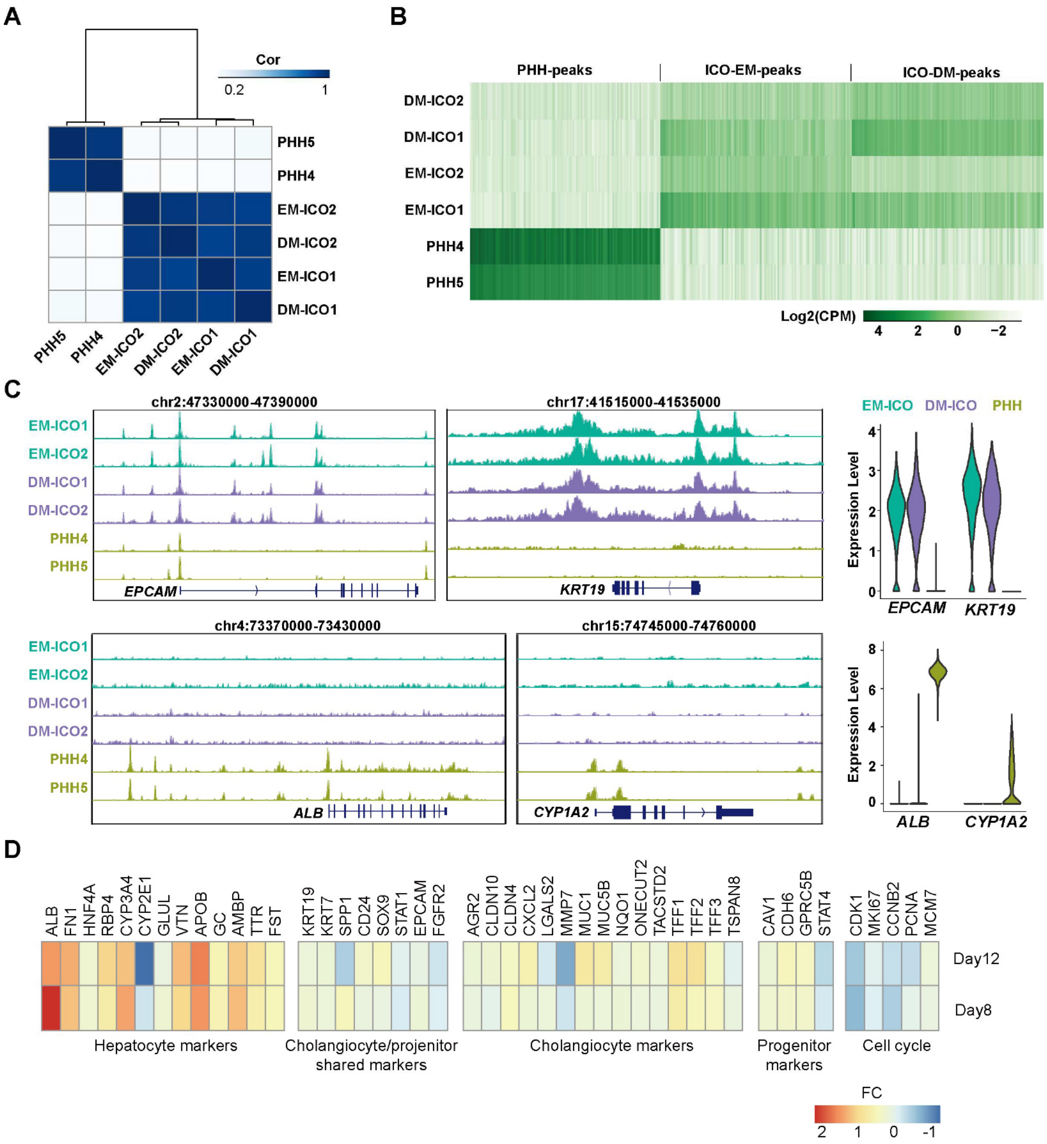

Fig. S5. Comparative analysis of PHHs and ICOs shows distinct chromatin profiles, and identified ELF3 as a barrier of hepatic differentiation in ICOs.

(**A**) heatmap of spearman correlation matrix of normalized open chromatin intensities in PHHs, EM-ICOs, and DM-ICOs identified by ATAC-seq. (**B**) Heatmap showing the difference of chromatin accessibility (top 2000 ATAC peaks of each sample) between PHHs and ICOs in EM and DM conditions. Data was shown in Log2(CPM). (**C**) Genome browser screenshot showing the chromatin state at *EPCAM*, *KRT19*, *ALB*, and *CYP1A2* genomic loci on the left, and the violin plots showing the corresponding expression levels in PHHs and ICOs on the

Disease Models & Mechanisms • Supplementary information

right. (**D**) Heatmap showing expression levels of hepatocyte markers, cholangiocyte/progenitor shared markers, cholangiocyte and progenitor unique genes identified from previous study as well as cell cycle related genes in siRNA transfected DM-ICOs (n=1) at day 8 (3 days post transfection) and day 12 (7 days post transfection). Data is shown as fold change (FC, siELF3/siCon).

Table S1. scRNA differentially expressed genes in each cluster

Available for download at
https://journals.biologists.com/dmm/article-lookup/doi/10.1242/dmm.050883#supplementary-data

Table S2. Motifs enriched in EM-ICOs and DM-ICOs

**Top10 Motifs enriched in EM-ICOs**					
Motifs	Factors	%input	%background	-log (p-value)	ROC AUC
GM.5.0.C2H2_ZF.0024	CTCF	11	<1	inf	0.58
GM.5.0.bZIP.0035	BACH2	10	<1	285.42	0.62
GM.5.0.bZIP.0013	JUN	10	<1	284.06	0.62
GM.5.0.bZIP.0058	NFE2L2	4	<1	84.87	0.59
GM.5.0.Nuclear_receptor.0028	HNF4A	4	<1	74.36	0.53
GM.5.0.Ets.0026	ELF3	3	<1	50.05	0.59
GM.5.0.CBF_NF-Y.0002	NFYA	3	<1	48.22	0.55
GM.5.0.Forkhead.0001	FOXI1	3	<1	43.75	0.54
GM.5.0.Forkhead.0008	FOXA1	3	<1	42.29	0.57
GM.5.0.Unknown.0004	NF1	3	<1	40.11	0.49
**Top10 Motifs enriched in DM-ICOs**					
Motifs	Factors	%input	%background	-log (p-value)	ROC AUC
GM.5.0.C2H2_ZF.0024	CTCF	11	<1	inf	0.58
GM.5.0.bZIP.0013	JUN	9	<1	250.29	0.59
GM.5.0.bZIP.0035	BACH2	7	<1	178.6	0.59
GM.5.0.Ets.0026	ELF3	3	<1	57.15	0.61
GM.5.0.Unknown.0004	NF1	3	<1	56.99	0.51
GM.5.0.Nuclear_receptor.0056	HNF4A	3	<1	55.65	0.53
GM.5.0.Forkhead.0008	FOXA1	3	<1	39.85	0.57
GM.5.0.C2H2_ZF.0288	SP6	2	<1	29.3	0.55
GM.5.0.CBF_NF-Y.0002	NFYA	2	<1	27.87	0.55
GM.5.0.C2H2_ZF.0314	KLF2	2	<1	27.02	0.55

Table S3. Patient information

Patient ID	Date of operation	Age	Sex	usage in this manuscript	Type of surgery
PHH1	2020/9/30	48	Male	scRNA-seq	Wedge resection segment V
PHH2	2020/10/1	72	Female	scRNA-seq	Wedge resection segment VI
PHH3	2020/10/14	76	Male	scRNA-seq	Segment VI/VII/VIII and wedge III resection
PHH4	2021/3/31	74	Male	bulk ATAC-seq	Segment VII resection
PHH5	2021/4/14	38	Male	bulk ATAC-seq	Wedge resection Segment VIII

Table S4. Primers used for qPCR

GLUL-qPCR-F1	TGGGAGCAGACAGAGCCTAT
GLUL-qPCR-R1	GATGCAAGATGAAACGGGCC
CYP2E1-qPCR-F1	CCTGCTCGTGGAAATGGAGA
CYP2E1-qPCR-R1	TCTCTGTCCCCGCAAAGAAC
OAT-qPCR-F1	TCTCCAGTTCCACAGACCCA
OAT-qPCR-R1	TACCATGAACGCAGCCACAT
CYP3A4-qPCR-F1	AAGTGGACCCAGAAACTGCA
CYP3A4-qPCR-R1	ACTTACGGTGCCATCCCTTG
CYP1A2-qPCR-F1	CACAGTCACCACAGCCATCT
CYP1A2-qPCR-R1	GGGCAAGAAGGAGGAGTGTC
ALB_qPCR_F	CTGCCTGCCTGTTGCCAAAGC
ALB_qPCR_R	GGCAAGGTCCGCCCTGTCATC
LGR5_qPCR_F	GACTTTAACTGGAGCACAGA
LGR5_qPCR_R	AGCTTTATTAGGGATGGCAA
GC-qPCR-F1	GCCTTGGTGAATGCTGTGAT
GC-qPCR-R1	TTGCTTTTAGTCGCTCTGCC
TTR-qPCR-F1	GAAAGGCTGCTGATGACACC
TTR-qPCR-R1	TACCACCTCTGCATGCTCAT
GAPDH-qPCR-F1	GCATCTTCTTTTGCGTCG
GAPDH-qPCR-R1	TGTAAACCATGTAGTTGAG

Disease Models & Mechanisms • Supplementary information

Table S5. ChIP-seq datasets reanalyzed in this study

EGR1-liver ChIP-seq	ENCODE Project Consortium	ENCFF389LQC, ENCFF132PDR
JUND-liver ChIP-seq	ENCODE Project Consortium	ENCFF215GBK, ENCFF978CPC
ATF3-liver ChIP-seq	ENCODE Project Consortium	ENCFF522PUA, ENCFF094LXX
HNF4A-liver ChIP-seq	ENCODE Project Consortium	ENCFF302XOK, ENCFF500ZBE
FOXA1-liver ChIP-seq	ENCODE Project Consortium	ENCFF765EAP, ENCFF945VNK
CTCF-liver ChIP-seq	ENCODE Project Consortium	ENCFF002EXB
RAD21-liver ChIP-seq	ENCODE Project Consortium	ENCFF643ZXX, ENCFF171UDL
EGR1- K562 ChIP-seq	ENCODE Project Consortium	ENCFF000PZK, ENCFF000PZP
JUND- K562 ChIP-seq	ENCODE Project Consortium	ENCFF000YSC, ENCFF000YSE
ATF3- K562 ChIP-seq	ENCODE Project Consortium	ENCFF000PWC, ENCFF000PWA
